# Antitumor Activity of Chinese Propolis in Human Breast Cancer MCF-7 and MDA-MB-231 Cells

**DOI:** 10.1155/2014/280120

**Published:** 2014-05-22

**Authors:** Hongzhuan Xuan, Zhen Li, Haiyue Yan, Qing Sang, Kai Wang, Qingtao He, Yuanjun Wang, Fuliang Hu

**Affiliations:** ^1^School of Life Science, Liaocheng University, Liaocheng 252059, China; ^2^School of Life Science, Anhui University, Hefei 230039, China; ^3^College of Animal Sciences, Zhejiang University, Hangzhou 310029, China

## Abstract

Chinese propolis has been reported to possess various biological activities such as antitumor. In present study, anticancer activity of ethanol extract of Chinese propolis (EECP) at 25, 50, 100, and 200 **μ**g/mL was explored by testing the cytotoxicity in MCF-7 (human breast cancer ER(+)) and MDA-MB-231 (human breast cancer ER(−)) cells. EECP revealed a dose- and time-dependent cytotoxic effect. Furthermore, annexin A7 (ANXA7), p53, nuclear factor-**κ**B p65 (NF-**κ**B p65), reactive oxygen species (ROS) levels, and mitochondrial membrane potential were investigated. Our data indicated that treatment of EECP for 24 and 48 h induced both cells apoptosis obviously. Exposure to EECP significantly increased ANXA7 expression and ROS level, and NF-**κ**B p65 level and mitochondrial membrane potential were depressed by EECP dramatically. The effects of EECP on p53 level were different in MCF-7 and MDA-MB-231 cells, which indicated that EECP exerted its antitumor effects in MCF-7 and MDA-MB-231 cells by inducing apoptosis, regulating the levels of ANXA7, p53, and NF-**κ**B p65, upregulating intracellular ROS, and decreasing mitochondrial membrane potential. Interestingly, EECP had little or small cytotoxicity on normal human umbilical vein endothelial cells (HUVECs). These results suggest that EECP is a potential alternative agent on breast cancer treatment.

## 1. Introduction

Propolis, a natural product and healthy food raw material, is collected by honeybees from various plants. It has been used since ancient times for its widely biological activities such as antibacterial, antiviral, antioxidant, anti-inflammatory, and antitumor [1, 2].

There are different types of propolis according to the location of its botanical origin and collecting season [3]. Based on Bankova classification, there are six main types of propolis, that is, poplar propolis, birch propolis, Brazilian green propolis, red propolis, pacific propolis, and Canarian propolis [4]. Chinese propolis is mainly classified as poplar-type and the predominant chemical compositions are flavonoids and phenolic compounds [5]. It exerts various biological activities including antitumor.

Recently, antitumor properties of propolis, the most remarkable activity, have been widely documented in various culture cell lines such as human leukemia (HL-60, CI41, U937), human colon cancer cells (SW480, HCT116), human cervical cancer (ME180, Hela), human glioblastoma cells (U87MG), human lung carcinoma (A549), human hepatocellular carcinoma (HepG2, SNU449), human pancreatic cancer (PANC-1, BxPC-3) cells, and mammary carcinoma (MCF-7) [6–15]. However, the molecular mechanisms of Chinese propolis on antitumor effects have not been fully elucidated and need to be deeply clarified. Here we investigated the anticancer effects of Chinese propolis in MCF-7 (human breast cancer ER(+)) and MDA-MB-231 (human breast cancer ER (−)) cells and then studied the underlying molecular mechanisms of Chinese propolis on both breast cancer cells.

## 2. Materials and Methods

### 2.1. Materials

DMEM was obtained from Gibco BRL Co. (USA). Fetal bovine serum was from Hyclone Laboratories Inc. (USA). Acridine orange (AO) was from Amresco (USA). Hoechst 33258, 2′,7′-dichlorodihydrofluorescin (DCHF), JC-1, propidium iodide (PI), and sulforhodamine B (SRB) were from Sigma Co. (USA). The antibodies against p53, ANXA7, NF-*κ*B p65, *β*-actin, and horseradish peroxidase-conjugated secondary antibodies were from Santa Cruz Biotechnology (USA). Secondary antibodies for immunofluorescence, donkey anti-rabbit IgG Alexa Fluor-488, were purchased from Life Technologies (USA). All the other reagents were of ultrapure grade.

### 2.2. Preparation of Propolis Extracts

Propolis used in the present study was Chinese propolis and had been used in our previous study [16]. The main plant origin was poplar (*Populus* sp.). Samples were maintained at −20°C before processing. Propolis sample was extracted with 95% ethanol at room temperature for 24 h. The ethanol suspension was filtered under reduced pressure. The filter liquid was then concentrated in a rotary evaporator under reduced pressure at 40°C until it reached a constant weight and was later redissolved in ethanol. The ethanol-extracted Chinese propolis (EECP) had a brown color. The prepared propolis was stored under a dry condition at 4°C.

To determine the constituents in EECP, we used high-performance liquid chromatography (HPLC) analysis with a photodiode array (PDA). EECP was dissolved in ethanol (2 mg/mL) to the injection of 10 *μ*L into the HPLC system. The HPLC system used was Agilent 1100 (Germany) with a C18 column (4.6 × 250 mm i.d., 5 *μ*m) at 28°C with a detection wavelength at 280 nm. The mobile phases consisted of methanol (A) and 0.1% phosphoric acid (B) at a flow rate of 1.0 mL/min.

### 2.3. Cell Cultures

The human breast cancer cells, MCF-7 and MDA-MB-231, were purchased from American Type Culture Collection (ATCC, USA). Human umbilical vein endothelial cells (HUVECs) were gifted by Atherosclerosis Research Institute of Taishan Medical University of China and purchased from ATCC. All cells were, respectively, cultured in DMEM medium, supplemented with 10% (v/v) FBS and 100 U/mL of penicillin, 100 *μ*g/mL streptomycin at 37°C under humidified 95–5% (v/v) air and CO_2_.

### 2.4. Cell Viability Assay

Cells were seeded at the density of 4 × 10^4^/mL into 96-well cell culture plates and were treated with different concentrations of EECP (25, 50, 100, and 200 *μ*g/mL). At 24 and 48 h, cells were precipitated for 1 h at 4°C with 100 *μ*L 10% trichloroacetic acid and stained with SRB. The optical density was measured at 492 nm after reconstitution of the dye in 100 *μ*L 10 mM Tris base. The viability (%) was expressed as (OD of treated group/OD of control group) ×100%. The viability of the control cells was set to 100%.

### 2.5. Nuclear Fragmentation Assay

Nuclear fragmentation was detected by acridine orange staining. Briefly, at 48 h, cells were stained with 5 *μ*g/mL AO at room temperature for 5 min and then were observed under a TE2000S fluorescence microscope (Nikon, Japan).

### 2.6. Hoechst 33258 Staining

Hoechst 33258 staining was used to observe apoptotic morphology. At 48 h, cells in all groups were stained with 10 *μ*g/mL Hoechst 33258 for 15 min. Cells were gently washed with PBS once. Nuclear condensation and fragmentation were observed under a TE2000S fluorescence microscope (Nikon, Japan).

### 2.7. Wound-Healing Assay

Cells were grown to 80% confluence in a 24-well plate. The monolayers were scratched with a plastic tip, washed by PBS to remove floating cell debris, and then incubated in medium in the absence or presence of different concentration of EECP for 48 h. Cell migration into the wound surface was determined under a TE2000S inverted microscope (Nikon, Japan). Migrated cells across the scratched lines were counted by Image-Pro Plus software (USA).

### 2.8. Immunofluorescence Microscopy Assay

As described in the previous report [17], MCF-7 and MDA-MB-231 cells treated with EECP were fixed in 4% paraformaldehyde (w/v) for 15 min at room temperature and blocked in 5% donkey serum (v/v) and then incubated with primary antibodies overnight at 4°C. Then cells were rinsed with 0.1 M PBS three times and incubated with corresponding FITC-conjugated secondary antibodies 1 h at 37°C. Cells were then rinsed three times with 0.1 M PBS to eliminate the uncombined secondary antibody. Nuclei were counterstained with PI. A laser scanning confocal microscope (Olympus FV1200, Japan) was used for fluorescence detection. Analysis was made by the Image-Pro Plus software (USA). Images were representative of three independent experiments.

### 2.9. Western Blot Assay

After treatment with EECP, cells were lysed in lysis buffer at ice. Thirty micrograms of protein were separated by 12% SDS-PAGE and transferred onto polyvinylidene difluoride (PVDF) membrane. The membrane was blocked with 5% (w/v) nonfat dry milk in PBS-Tween 20 (PBST; 0.05%) for 1 h and was incubated with primary antibody (1 : 1,000 in PBST) at 4°C overnight. After three washings in PBST, the PVDF membrane was incubated with appropriate horseradish peroxidase-conjugated secondary antibodies (1 : 5,000) for 1 h at room temperature. The immunoreactive bands were developed with ECL western blotting system. The relative quantity of proteins was analyzed by use of Quantity One software (Bio-Rad, Hercules, CA, USA).

### 2.10. Determination of Intracellular ROS Levels

Intracellular ROS level was measured with 2′,7′-dichlorodihydrofluorescein (DCHF), which could be rapidly oxidized into the highly fluorescent 2′,7′-dichlorofluorescein (DCF) in the presence of intracellular ROS [18]. We treated cells as mentioned above for 48 h then washed cells with basal DMEM medium for 5 min and incubated the cells with DCHF 1 mL at 37°C for 30 min. After washing the cells three times with basal DMEM medium, the fluorescence was monitored with a confocal laser scanning microscope (Olympus FV1200, Japan) using excitation and emission wavelengths of 488 nm. The amount of ROS was quantified by Image-Pro Plus software (USA). The images were representative of three independent experiments.

### 2.11. Intracellular Mitochondrial Membrane Potential Assay

Fluorescence probe of JC-1 was used to test mitochondrial membrane potential. JC-1 exists as a monomer at low mitochondrial membrane potential and emits green fluorescence but forms aggregates and emits red fluorescence at high mitochondrial membrane potential [19]. Cells were treated for 48 h then washed with basal DMEM medium for 5 min and incubated with JC-1 1 mL at 37°C for 15 min. After washing the cells three times with basal DMEM medium, the fluorescence was monitored with a confocal laser scanning microscope (Olympus FV1200, Japan) using excitation and emission wavelengths of 488 and 546 nm, respectively. Results were shown by ratio of red to green fluorescence as compared with the control; Image-Pro Plus software (USA) was used to analysis fluorescence intensity.

### 2.12. Statistical Analysis

Data are from at least three independent experiments and expressed as means ± S.E.M. Statistical analysis involved the paired Student *t* test and ANOVA with SPSS version 11.5. Differences were considered statistically significant at *P* < 0.05.

## 3. Results

### 3.1. Constituents of EECP

**Figure 1:**
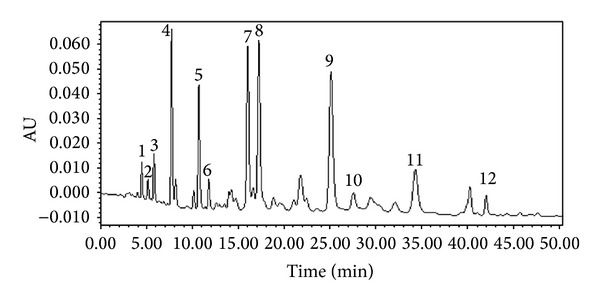
The constituents from ethanol-extracted Chinese propolis (EECP). 1. Caffeic acid; 2.* p*-coumaric acid; 3. ferulic acid 4. pinobanksin; 5. 7-hydroxy-5-methoxyflavanone; 6. kaempferol; 7. Pinocembrin; 8. pinobanksin 3-acetate; 9. chrysin; 10. caffeic acid phenethyl ester; 11. galangin; 12. tectochrysin.

The main constituents identified in our sample are caffeic acid,* p*-coumaric acid, ferulic acid, pinobanksin, 7-hydroxy-5-methoxyflavanone, kaempferol, pinocembrin, pinobanksin 3-acetate, chrysin, caffeic acid phenethyl ester, galangin, and tectochrysin by HPLC analysis (Figure 1).

### 3.2. EECP Decreased MCF-7 and MDA-MB-231 Cells Proliferation but Had Little Effect on Normal HUVECs

**Figure 2:**
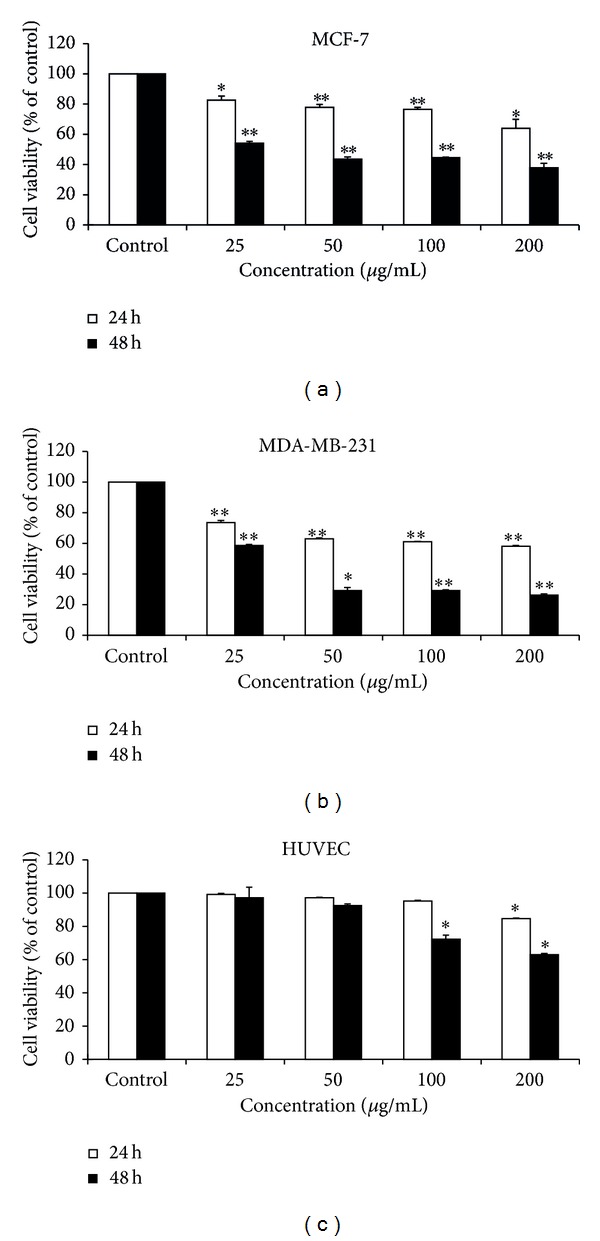
EECP decreased MCF-7 and MDA-MB-231 cells proliferation but had little/small effect on normal HUVECs. (a) EECP inhibited MCF-7 proliferation at 24 and 48 h. (b) EECP inhibited MDA-MB-231 cells proliferation at 24 and 48 h. (c) EECP had little effect on normal HUVECs viability under the concentration of 100 *μ*g/mL (*P < 0.05, **P < 0.01 versus control, *n* = 3). Data are means ± S.E.M.

We investigated the sensitivity of MCF-7 and MDA-MB-231 cells to EECP (25, 50, 100, and 200 *μ*g/mL) using SRB assay at 24 and 48 h. EECP significantly inhibited MCF-7 and MDA-MB-231 cells proliferation in a dose- and time-dependent manner. Notably, the inhibitory effect of EECP on MDA-MB-231 cells was higher than on MCF-7 cells (*P < 0.05, **P < 0.01; Figures 2(a) and 2(b)).

We also investigated the effect of EECP on normal HUVECs viability, and the results showed that there was little effect on proliferation of normal HUVECs under the concentrations of 100 *μ*g/mL. However, EECP at concentration of 200 *μ*g/mL had small cytotoxicity (*P < 0.05; Figure 2(c)).

### 3.3. EECP Induced Apoptosis in MCF-7 and MDA-MB-231 Cells

**Figure 3:**
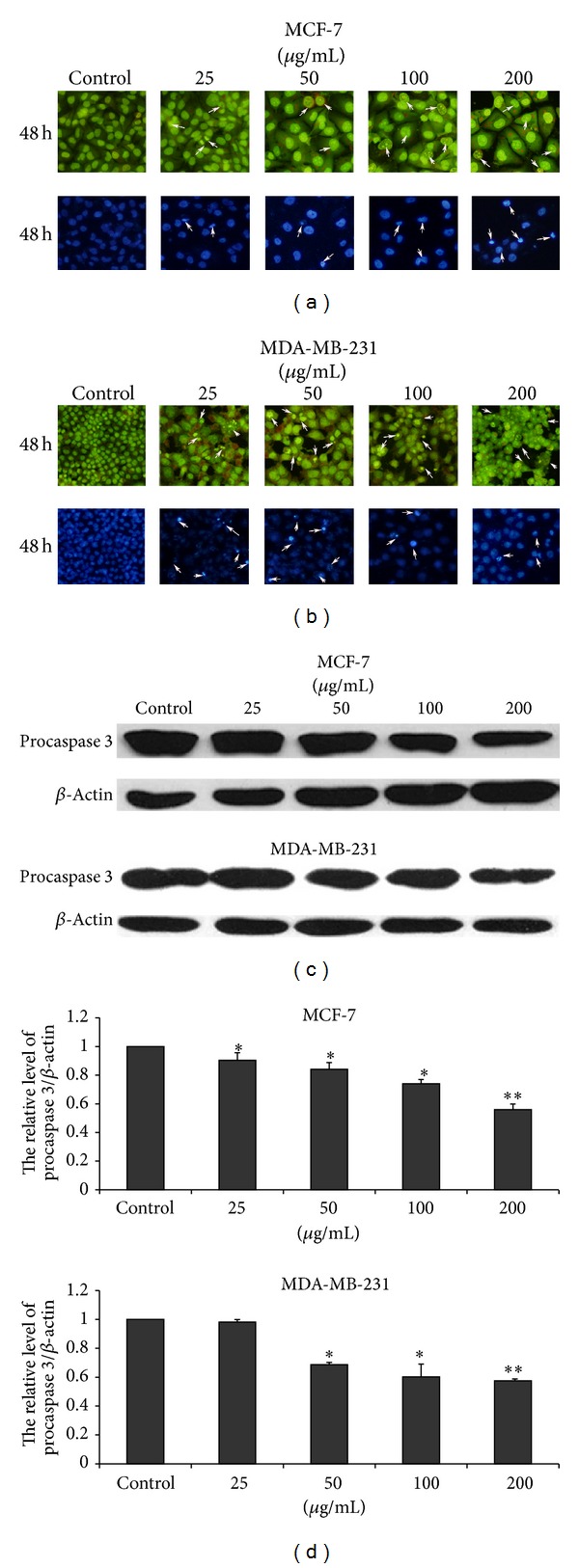
EECP induced apoptosis in MCF-7 and MDA-MB-231 cells. (a) Morphological changes of nuclei in MCF-7 cells by staining with acridine orange and Hoechst 33258 at 48 h (×200). (b) Morphological changes of nuclei in MDA-MB-231 cells by staining with acridine orange and Hoechst 33258 at 48 h (×200). (c) The levels of procaspase 3 (35KD) were detected by western blot at 24 h. (d) The hemiquantification of procaspase 3 levels in MCF-7 and MDA-MB-231 cells (*P < 0.05, **P < 0.01 versus control, *n* = 3).

Acridine orange and Hoechst 33258 staining results indicated that different concentrations of EECP evidently induced nuclear condensation and fragmentation of MCF-7 and MDA-MB-231 cells in a dose-dependent manner. Importantly, nuclei of MDA-MB-231 cells treated with EECP 200 *μ*g/mL were almost fragmentation (Figures 3(a) and 3(b)). In addition, we also tested procaspase 3 (35KD) by western blot at 24 h. The results showed that caspase 3 was activated in MCF-7 and MDA-MB-231 cells treated with EECP (Figures 3(c) and 3(d)).

### 3.4. EECP Inhibited MDA-MB-231 Cells Migration

**Figure 4:**
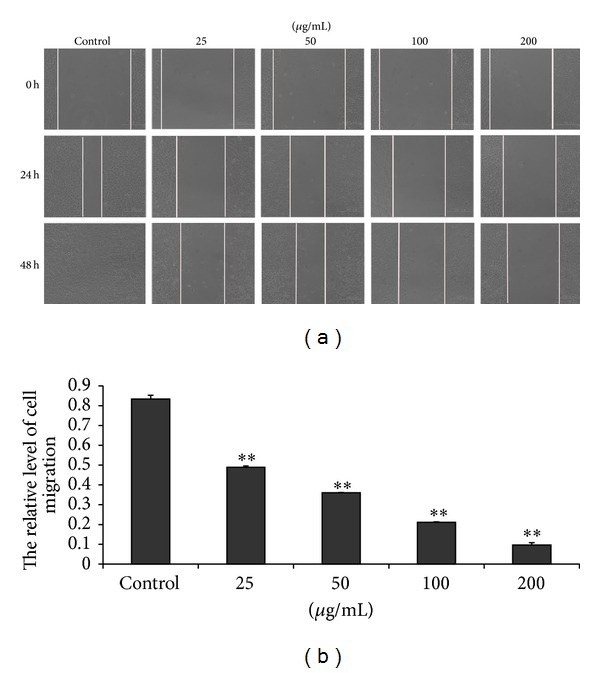
EECP inhibited MDA-MB-231 cells migration. (a) Cell migration micrographs obtained under a phase contrast microscope at 0, 24, and 48 h (×100). (b) Relative levels of cell migration (**P < 0.01 versus control, *n* = 3).

Roughly 70% of all patients dying of breast cancer have bone metastases [20]. Therefore, we also performed wounding-healing experiments to detect the effect of EECP on migration of MCF-7 and MDA-MB-231 cells. EECP significantly inhibited MDA-MB-231 cells migration in a dose-dependent manner at 48 h (**P < 0.01; Figure 4), whereas inhibitory effect of EECP on migration of MCF-7 cells was not significant (data were not shown).

### 3.5. EECP Regulated the Levels of ANXA7, p53, and NF-*κ*B p65 in MCF-7 and MDA-MB-231 Cells

**Figure 5:**
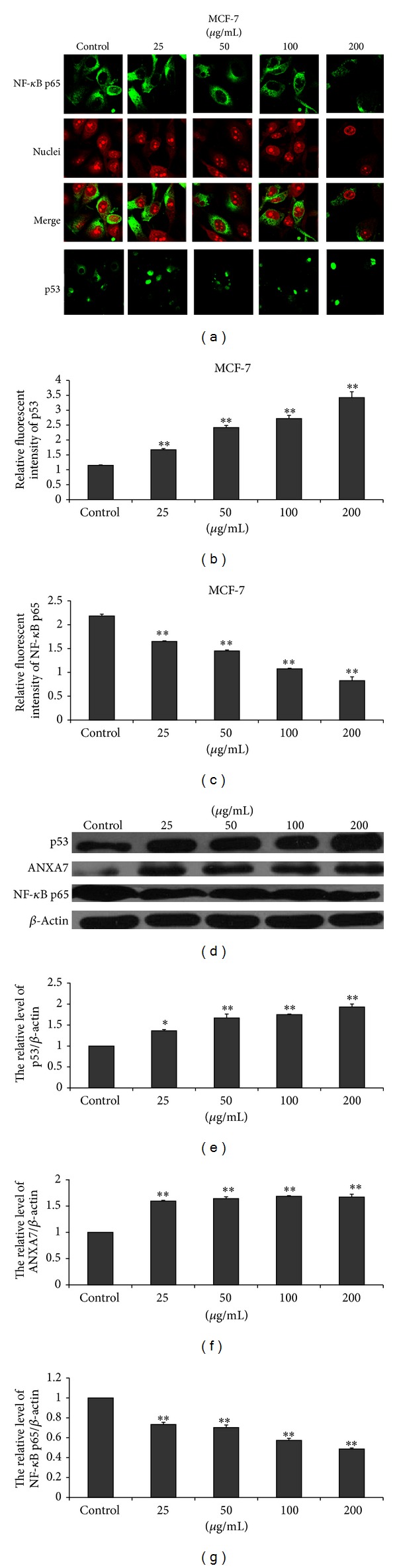
EECP regulated the levels of ANXA7, p53, and NF-*κ*B p65 in MCF-7 cells. (a) Fluorescent micrographs obtained at 48 h (×400). Nuclei were counterstained with PI. (b) and (c) The relative fluorescence intensity of NF-*κ*B p65 and p53 in MCF-7 cells. (d) The levels of p53, ANXA7, and NF-*κ*B p65 were detected by western blot at 48 h. (e), (f), and (g) The hemiquantification of p53, ANXA7, and NF-*κ*B p65 levels in MCF-7 cells (*P < 0.05, **P < 0.01 versus control, *n* = 3).

**Figure 6:**
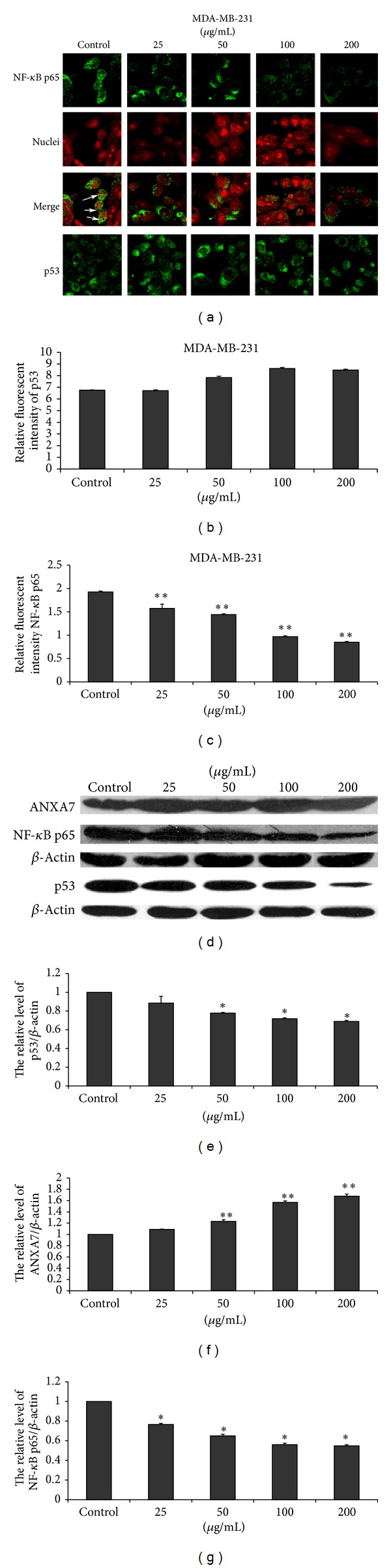
EECP regulated the levels of ANXA7, p53, and NF-*κ*B p65 in MDA-MB-231 cells. (a) Fluorescent micrographs obtained at 48 h (×400). (b) and (c) The relative fluorescence intensity of NF-*κ*B p65 and p53 in MDA-MB-231 cells. (d) The levels of ANXA7 and NF-*κ*B p65 were detected by western blot at 48 h; the level of p53 was detected by western blot at 24 h. (e), (f), and (g) The hemiquantification of p53, ANXA7, and NF-*κ*B p65 levels in MDA-MB-231 cells (*P < 0.05, **P < 0.01 versus control, *n* = 3).

EECP significantly upregulated the expression of ANXA7 and downregulated NF-*κ*B p65 level in a dose-dependent manner by western blot and immunofluorescent assay in MCF-7 and MDA-MB-231 cells. Furthermore, the translocation of NF-*κ*B p65 from cytoplasm to nuclei was also inhibited by EECP in both cells. The effect of EECP on p53 level in MCF-7 and in MDA-MB-231 cells was different. p53 level was significantly increased in MCF-7 cells. However, in MDA-MB-231 cells, EECP evidently inhibited p53 level (*P < 0.05, **P < 0.01; Figures 5 and 6).

### 3.6. EECP Increased ROS Level in MCF-7 and MDA-MB-231 Cells

**Figure 7:**
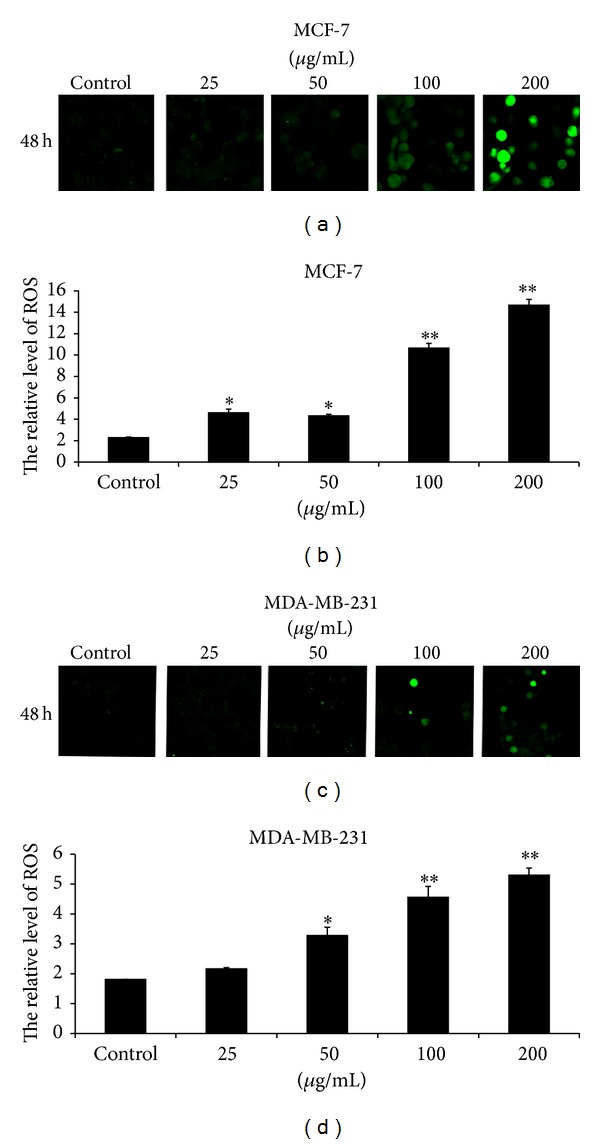
EECP increased ROS level in MCF-7 and MDA-MB-231 cells. (a) Fluorescent micrographs of MCF-7 cells obtained at 48 h (×200). (b) Relative quantity of ROS in MCF-7 cells. (c) Fluorescent micrographs of MDA-MB-231 cells obtained at 48 h (×200). (d) Relative quantity of ROS in MDA-MB-231 cells. Values represent the relative fluorescent intensity determined by laser scanning confocal microscopy (*P < 0.05, **P < 0.01 versus control, *n* = 3).

EECP at concentration of 25–200 *μ*g/mL significantly increased ROS level in MCF-7 cells, whereas, in MDA-MB-231 cells, EECP at concentration of 50–200 *μ*g/mL evidently increased ROS level (*P < 0.05, **P < 0.01; Figure 7).

### 3.7. EECP Reduced Mitochondrial Membrane Potential in MCF-7 and MDA-MB-231 Cells

**Figure 8:**
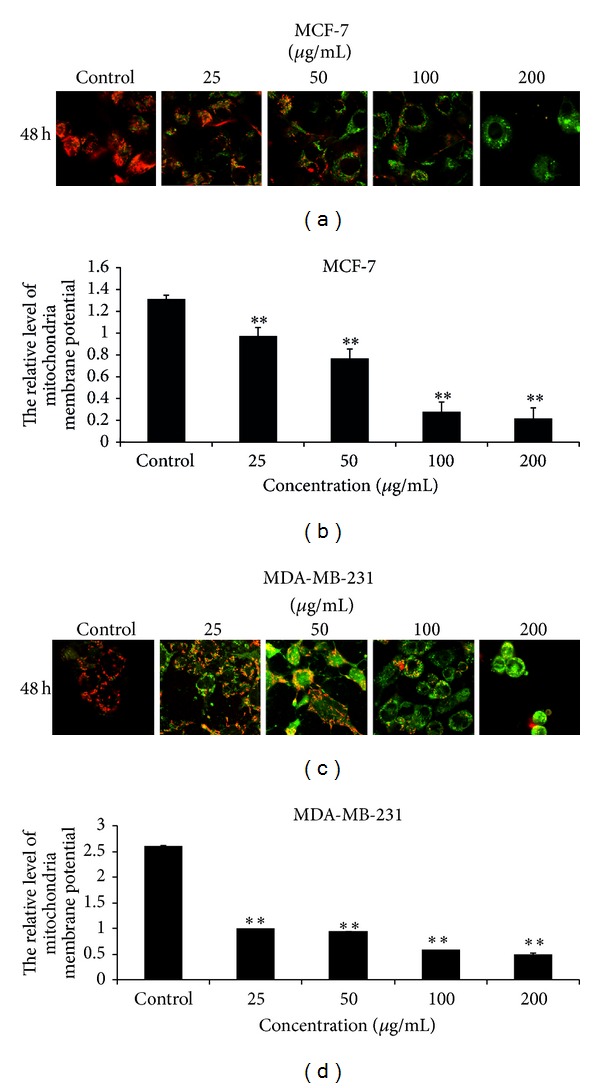
EECP reduced mitochondrial membrane potential in MCF-7 and MDA-MB-231 cells. (a) Fluorescent micrographs of MCF-7 cells obtained at 48 h (×400). (b) Relative quantity of mitochondrial membrane potential in MCF-7 cells. (c) Fluorescent micrographs of MDA-MB-231 cells obtained at 48 h (×400). (d) Relative quantity of mitochondrial membrane potential in MDA-MB-231 cells. Values represent the relative fluorescent intensity determined by laser scanning confocal microscopy (**P < 0.01 versus control, *n* = 3).

The mitochondrial membrane potential sensor JC-1 was used to determine the mitochondrial function. As shown in Figure 7, EECP significantly decreased mitochondrial membrane potential in a dose-dependent manner in MCF-7 and MDA-MB-231 cells at 48 h. Note that the decreased level of mitochondrial membrane potential in MDA-MB-231 cells was higher than that in MCF-7 cells (**P < 0.01; Figure 8).

## 4. Discussion

In propolis, there is usually a variety of chemical compounds, such as polyphenols, terpenoids, steroids, and amino acid. Propolis samples obtained from different plants are composed of different chemical compounds. Chinese propolis is mainly classified as poplar-type and the predominant chemical constituents are flavonoids and phenolic compounds, and their percentage ranges from 35% to 50% [21]. Our results from HPLC also indicate that the major chemical constituents of EECP are polyphenolic/flavonoids. And caffeic acid phenethyl ester, caffeic acid, galangin, chrysin, kaempferol, pinobanksin, and pinocembrin are the major compounds. Furthermore, accumulating evidence has indicated that polyphenolic/flavonoids may serve as a potent adjunct to chemotherapy and radiotherapy in the treatment of cancers [22–24].

Breast cancer ranks among the most common malignant tumors afflicting women worldwide [25]. In this study we investigated the antitumor activities of EECP in MCF-7 and MDA-MB-231 cells. Our results showed that EECP potentially exerted its antitumor effect by inhibiting cell proliferation, inducing apoptosis, inhibiting cell migration, regulating ANXA7 and p53 levels, downregulating NF-*κ*B p65 level and inhibiting its translocation from cytoplasm to nuclei, and increasing intracellular ROS level, decreasing mitochondrial membrane potential. Besides these, EECP had little or small effect on normal HUVECs. Interestingly, MDA-MB-231 cells were more sensitive to EECP than MCF-7 cells.

The vascular endothelium cells play a critical role in the physiological and pathological progress for their location between the intravascular compartment and extravascular tissues [17]. Endothelial cells are the primary target for many chemical agents. Many anticancer chemical agents cannot be used in clinic for their cytotoxicity on endothelial cells. Here we found that EECP under concentration 100 *μ*g/mL had good antitumor activity but had little cytotoxicity on normal HUVECs, and concentration of EECP 200 *μ*g/mL had some small toxicity on HUVECs, which indicated the low toxicity of EECP when used as anticancer agent.

Cancer metastasis is the leading cause of mortality in patients with breast cancer. Metastasis is multistep process. MDA-MB-231 breast cancer cells, a highly metastatic human breast carcinoma cell line, are widely used as a model to study breast cancer cell metastasis. Here we found that EECP 25–200 *μ*g/mL remarkably inhibited MDA-MB-231 cells migration, which indicated the good ability of EECP on inhibiting breast cancer cells metastasis.

ANXA7, a member of the annexin family of calcium-dependent phospholipid binding proteins, codes for Ca^2+^ dependent GTPase, which involves several different roles in autophagy, exocytosis, carcinogenesis, and tumor suppression [26–28]. Recently, it is described as a candidate tumor suppressor gene for prostate cancer [29]. Human ANXA7 has been mapped to tumor susceptibility locus 10q21 with 35% loss of heterozygosity in prostate and breast cancer indicating its possible tumor suppressive function [30]. Srivastava et al. (2001) indicated that ANXA7 could be a biomarker in the progression of breast cancer [31]. In present study, we found that EECP significantly upregulated ANXA7 level in MCF-7 and MDA-MB-231 cells. This is the first time indicating the effect of propolis on ANXA7 in breast cancer cells, which might be a new target of propolis on antitumor study and treatment.

p53, another tumor suppressor protein, is a central target of inactivation in human cancer and a key regulator of genotoxic stress-induced growth arrest or apoptosis [32]. We previously reported that both Chinese propolis and Brazilian green propolis affected p53 level in HUVECs with nutrition deprivation [16, 33]. MCF-7 has a wild-type p53; here we found that EECP higher 25 *μ*g/mL significantly upregulated p53 level to induce apoptosis. However, MDA-MB-231 has a high level of a mutant p53, which contributes to the suppression of apoptosis in human breast cancer cells. In current study, we found that the effect of EECP on mutant-type p53 was complex and was dose-dependent. p53 level was inhibited with the increase of concentration by western blot assay. However, these changes could not be tested by immunofluorescence microscopy assay at 24 h for there was a high level of mutant p53-expressing in MDA-MB-231. Taken together, EECP exerts its antitumor effect through regulating p53 level.

The NF-*κ*B signal transduction pathway is deregulated in a variety of human cancers [34, 35]. In most types of cancer cells, NF-*κ*B is constitutively active. Blocking NF-*κ*B has been shown to stop tumor cells from proliferating, to die, or to become more sensitive to the action of antitumor agents, especially antioxidants [36]. Therefore, agents capable of downmodulating the activation of NF-*κ*B have a potential for use in therapeutic interventions [37]. Here we found that EECP downregulated the activation of NF-*κ*B p65, a subunit of NF-*κ*B, and inhibited its translocation from cytoplasm to nuclear to activate in MCF-7 and MDA-MB-231 cells, which indicated that EECP could become a useful antitumor agent.

Accumulating evidence has demonstrated that ROS are important signals in the regulation of diverse cellular functions. High levels of ROS induce oxidative stress, leading to a number of different diseases, including cancer [38]. However, recent studies indicated that high levels ROS induce apoptosis by triggering proapoptotic signaling molecules to antitumor [39]. And we previously found that a high concentration of Brazilian propolis extract induced HUVECs apoptosis with ROS level increase, and at a low concentration propolis protected HUVECs by decreasing ROS level [33]. These findings taken together, it appears that propolis plays a dual role on ROS depending on concentrations: at high concentration, it exerts a prooxidant effect; at low concentration, it can also act as an antioxidant by scavenging free radicals. In present study, EECP induced MCF-7 and MDA-MB-231 cells apoptosis with ROS increase in a dose-dependent manner. EECP may therefore exert prooxidant effect in breast cancer cells.

The decrease of mitochondrial membrane potential would lead to the release of cytochrome c to activate caspase to initiate apoptotic signaling pathway [40]. Mitochondria are the most important intracellular source of ROS, and elevated ROS levels can also decrease mitochondrial membrane potential [33]. Here, we found that EECP decreased mitochondrial membrane potential in MCF-7 and MDA-MB-231 cells. From this viewpoint, we deduced that EECP induced apoptosis in MCF-7 and MDA-MB-231 cells were ROS-dependent mitochondrial pathway.

In conclusion, our results suggest that EECP and its polyphenolic/flavonoid components exert antitumor effects mainly through inducing apoptosis of breast cancer cells. The involved mechanisms commonly contain ANXA7 and p53 proteins regulating, NF-*κ*B inhibition, and regulation of ROS and mitochondrial membrane potential. Attractively, EECP has non-/low toxicity to normal cells because of its selective toxicities to tumor cells. So it is believed that propolis may become an attractive and promising agent for breast cancer treatment. However, further research is needed to clarify precise targets of propolis in breast cancer cells.
